# Design and Additive Manufacturing of a Biomimetic Customized Cranial Implant Based on Voronoi Diagram

**DOI:** 10.3389/fphys.2021.647923

**Published:** 2021-04-09

**Authors:** Neha Sharma, Daniel Ostas, Horatiu Rotar, Philipp Brantner, Florian Markus Thieringer

**Affiliations:** ^1^Clinic of Oral and Cranio-Maxillofacial Surgery, University Hospital Basel, Basel, Switzerland; ^2^Department of Biomedical Engineering, Medical Additive Manufacturing Research Group (SwissMAM), University of Basel, Allschwil, Switzerland; ^3^Department of Oral and Cranio-Maxillofacial Surgery, “Iuliu Hatieganu” University of Medicine and Pharmacy, Cluj-Napoca, Romania; ^4^Department of Radiology and Nuclear Medicine, University Hospital Basel, Basel, Switzerland

**Keywords:** biomimetics, computer-aided design, cranial reconstruction, patient-specific implant, selective laser melting, Voronoi diagram, additive manufacturing, 3D printing

## Abstract

Reconstruction of cranial defects is an arduous task for craniomaxillofacial surgeons. Additive manufacturing (AM) or three-dimensional (3D) printing of titanium patient-specific implants (PSIs) made its way into cranioplasty, improving the clinical outcomes in complex surgical procedures. There has been a significant interest within the medical community in redesigning implants based on natural analogies. This paper proposes a workflow to create a biomimetic patient-specific cranial prosthesis with an interconnected strut macrostructure mimicking bone trabeculae. The method implements an interactive generative design approach based on the Voronoi diagram or tessellations. Furthermore, the quasi-self-supporting fabrication feasibility of the biomimetic, lightweight titanium cranial prosthesis design is assessed using Selective Laser Melting (SLM) technology.

## Introduction

Cranial defects reconstruction is an arduous task for craniomaxillofacial surgeons. Secondary to head trauma, cerebral tumors, congenital defects, infections, or previous surgery complications, cranial reconstruction aims to restore the skull's integrity to ensure adequate protection and functionality of the underlying brain (Goiato et al., 2009; Aydin et al., 2011). Moreover, it alleviates the psychological and social consequences of esthetic impairment (Bonda et al., 2015; Alkhaibary et al., 2020).

Among all alloplastic materials, titanium continues to be the mainstream material used in cranioplasty due to its excellent biocompatibility, resistance to infection, high strength to weight ratio, corrosion resistance, non-magnetic properties, and toughness (Niinomi, 1998; Zhang and Chen, 2019). Titanium plates for cranial defect reconstructions were first described in 1974 (Gordon and Blair, 1974). Since then, cranial reconstructions have witnessed tremendous progress in using computer-aided design (CAD) methods (Cabraja et al., 2009; Wiggins et al., 2013; Bonda et al., 2015). Additive manufacturing (AM) or three-dimensional (3D) printing of titanium patient-specific implants (PSIs) made its way into cranioplasty, improving the clinical outcomes in complex surgical procedures (Cho et al., 2015; Park et al., 2016; Moiduddin et al., 2019; Sharma et al., 2020). Furthermore, there has been a significant interest within the medical community in redesigning implants based on natural analogies (Tejero et al., 2014; Brett et al., 2017).

Biomimicry is one of the most important research interests in modern manufacturing technologies and has further opened the way to significant medical device development improvements (Hwang et al., 2015). Biomimetics is the study of nature and natural phenomena to understand the principles of biological substances, structures, and processes to create products that mimic nature counterparts (Vincent, 2009; Schaedler and Carter, 2016; Jungck et al., 2019). In pursuit of biomimicry, the “Voronoi diagram”—a mathematical and fundamental geometrical construct, has been used to computationally reproduce many natural formations, including the complicated bone structure (Li et al., 2014; Vázquez-Otero et al., 2015). The “Voronoi diagram” has also been used for the conception of porous implants, with a focus on scaffolds for tissue engineering (Giannitelli et al., 2014; Fantini et al., 2016; Gómez et al., 2016; Chen et al., 2020). With the emergence and assimilation of CAD modeling and AM technologies, nature-inspired, biomimetic structures can now be designed and fabricated.

This paper proposes a workflow to create a biomimetic patient-specific cranial prosthesis with an interconnected strut macrostructure mimicking bone trabeculae, based on the Voronoi diagram or tessellations. Using an interactive CAD modeling approach, the authors aim to access the quasi-self-supporting fabrication feasibility of the cranial prosthesis designs with AM technology.

## Materials and Methods

In this section, the proposed methodology for designing and fabricating a biomimetic, lightweight patient-specific cranial prosthesis is described. The overall workflow was performed in three modules, as described in detail below.

### Preoperative Medical Image Data Acquisition and Processing

**Figure 1:**
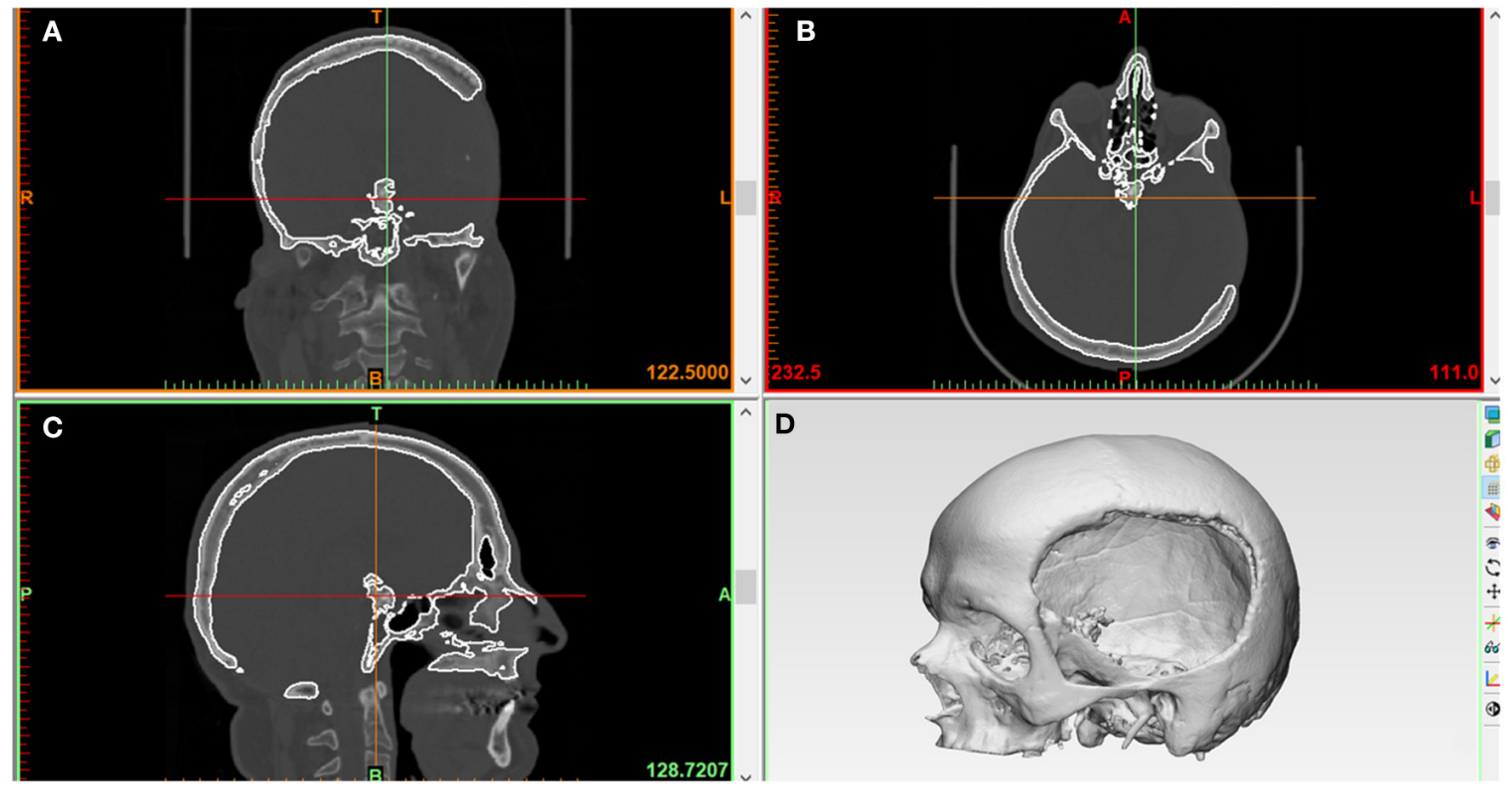
Medical image processing for the generation of a three-dimensional (3D) volumetric reconstruction of the patient's skull with unilateral cranial deformity. **(A)** Coronal view. **(B)** Axial view. **(C)** Sagittal view. **(D)** Skull 3D volumetric reconstruction.

An anonymized unilateral cranial defect case selected from the University Hospital Basel database was used for the workflow. The skull of a patient with a left fronto-temporo-parietal cranial deformity was scanned using a high-resolution computed tomography (CT) (Siemens SOMATOM, Siemens Healthcare GmbH, Erlangen, Germany) with the following parameters: (1) matrix of 512 × 512 pixels, (2) slice thickness of 1 mm, (3) seed per rotation of 1 mm, (4) gantry tilt 0°, and (5) bone window setting or high-resolution reconstruction algorithm. The Digital Imaging and Communications in Medicine (DICOM) dataset of the 2-dimensional (2D) image slices generated from the CT scan were imported into a medical image processing software (Mimics Innovation Suite v. 21.0, Materialise, Leuven, Belgium). Following this, threshold selection was made, in which the inbuilt greyscales were selected using bone-specific Hounsfield units (HU). A 3D volumetric reconstruction image of the skull anatomy was generated using the software-integrated semiautomatic segmentation and crop-mask tools. Figure 1 illustrates the accomplished steps of segmentation from a fully scanned skull to the desired region of interest, i.e., the 3D skull model (defect region) without the neck and mandible. The 3D skull image was saved in the standard tessellation language (.STL) file format for the subsequent cranial prosthesis modeling process.

### Three-Dimensional (3D) Modeling and Design Process of Patient-Specific Cranial Implants

**Figure 2:**
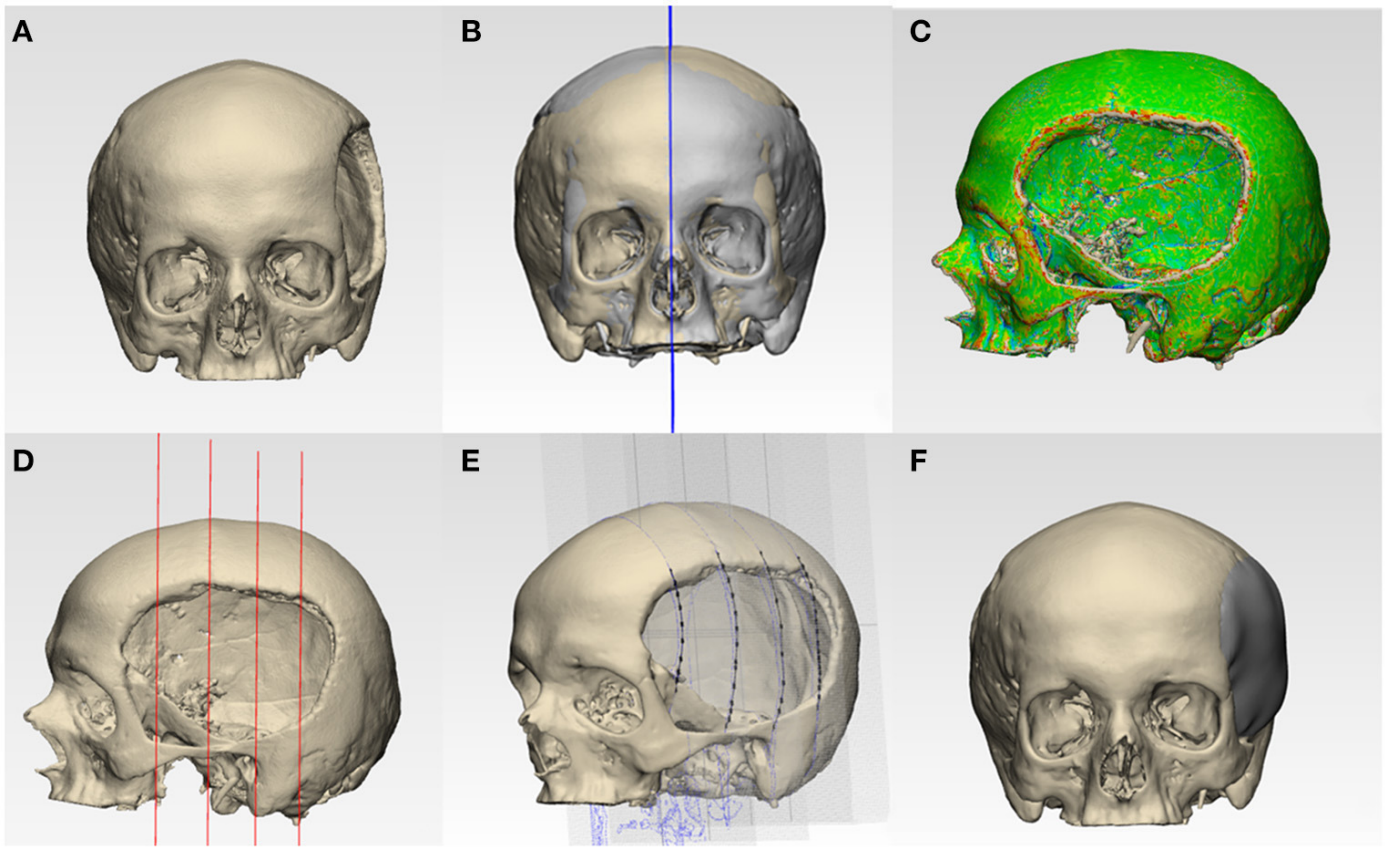
Schematic representation of steps involved in the anatomical design modeling phase for reconstructing a patient-specific cranial prosthesis. **(A)** Three-dimensional (3D) volumetric reconstruction of the skull model. **(B)** Creation of a datum plane and mirroring of the healthy side onto the defect side. **(C)** Curvature analysis and optimization of a curve. **(D)** Equidistantly spaced sketch graphs oriented perpendicular to the datum plane. **(E)** Spline creation by the importation of contour reference data from the curve, mirrored, and defect side. **(F)** Reconstructed skull with the cranial prosthesis.

This step's main objective was to design a biomimetic, lightweight patient-specific cranial prosthesis by incorporating two phases, i.e., anatomical and generative design modeling. In the anatomical design modeling phase, .STL file of the 3D volumetric reconstructed model (Figure 2A) was imported into a medically certified CAD modeling software (3-matic Medical v. 13.0, Materialise, Leuven, Belgium). For the precise reconstruction of the virtual cranial defect, a mirror-based reconstruction approach was followed (Benazzi and Senck, 2011; Chamo et al., 2020). First, a datum plane (mid-plane reference system) was created, and the healthy (non-defect) side was mirrored to the defect side. For accurate alignment and extraction of the contours for prosthesis reconstruction, the mirrored side was aligned with the surrounding bone on the defect side. Using a subsequent iterative closest point (ICP) protocol–a surface-based matching algorithm (Zhang, 1994; Benazzi and Senck, 2011) that minimizes the distance between 2-point clouds by the least-squares method–global registration was achieved (Figure 2B). Subsequently, skull curvature analysis was performed, designing a curve that outlines the defect margin (Figure 2C). To maintain tangency between the prosthesis and the skull bone, defining the curve close to the anatomical defect region in low curvature areas is essential. Subsequently, four equally spaced sketch graphs were oriented perpendicular to the datum plane at different positions in the skull model (Figure 2D). The contours extracted from the designed curve–healthy and mirrored skull model–were imported into the individual sketches, which acted as reference planes for prosthesis creation. The proposed method's main idea was to use these reference planes as anatomical constraints and guide the points for the thin-plate spline (TPS) algorithm (Figure 2E). The TPS algorithm interpolates and estimates the missing data based on analogous reference points present in the mirrored and defect skull model. Finally, using an internal coordinate system algorithm based on anatomically constrained deformation, the prosthesis geometry with a tapered edge overlapping the surrounding bone was generated. A “surface/triangle normal” or an inward-directed undercut operation was then performed on the prosthesis to remove any obstruction from fitting onto the skull model (Figure 2F). Finally, the customized prosthesis was saved and exported in an .STL file format for further modeling.

**Figure 3:**
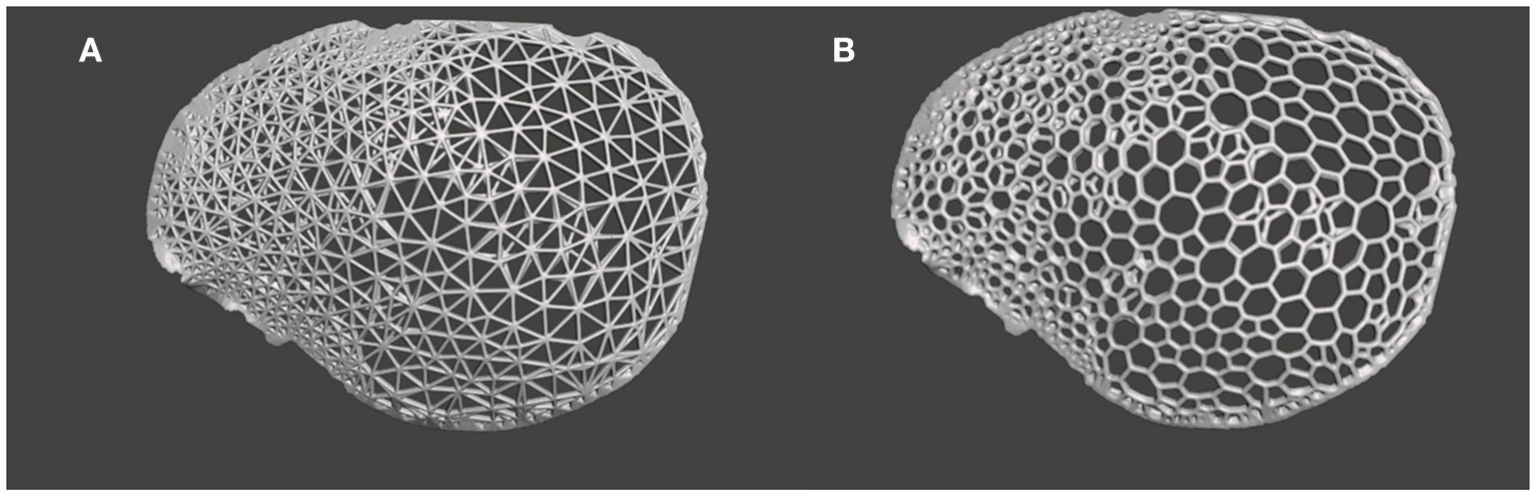
Generative design modeling phase for the generation of a biomimetic patient-specific cranial prosthesis. **(A)** Wireframe/lattice Voronoi prosthesis design generated using the “Mesh+Delaunay Edges” algorithm. **(B)** Organic Voronoi prosthesis design developed using the “Mesh+Delaunay Dual Edges” algorithm.

In the subsequent generative design modeling phase, surface modeling and generation of biomimetic macrostructures on the customized cranial prosthesis was accomplished based on the Voronoi tessellations algorithm. The .STL file of the cranial prosthesis was imported in free computer-aided modeling (CAM) software (Meshmixer, v. 3.5 for Windows, Autodesk Inc., San Rafael, California, USA). To achieve computational processability, the mesh simplification method was used. The digital prosthesis file (.STL) composing tens of thousands of triangles was remodeled using a shape-preserving command to reduce the number of edges or pairs of vertices. This adaptive mesh refinement was used on a defined region of interest and consisted of operations of edge contractions and edge flipping, generating new vertex positions. Subsequently, using “Pattern” functionality in the software, two design approaches were implemented. The “Mesh+Delaunay Edges” and the “Mesh+Delaunay Dual Edges” commands were executed on the cranial prosthesis .STL file. In both design commands, the global Voronoi tessellation algorithm was performed for all seed points. The respective commands finally processed the divided edges with corresponding strut thicknesses to generate a Voronoi based macrostructure consisting of interconnected struts with multiple holes/openings. A mesh Boolean intersection between the prosthesis geometry and the particular pattern was then used. Subsequently, these commands resulted in two designs for the cranial prosthesis: design 1- a wireframe/lattice Voronoi pattern and design 2- an organic, bone trabeculae-like Voronoi pattern. These commands were iteratively tested, and an element dimension of 2 mm was finally selected after wall/strut thickness analysis. Two biomimetic prosthesis designs with interconnected struts and multiple gradient holes/openings were generated, as shown in Figure 3.

### Topographic Bone Thickness Map Generation

**Figure 4:**
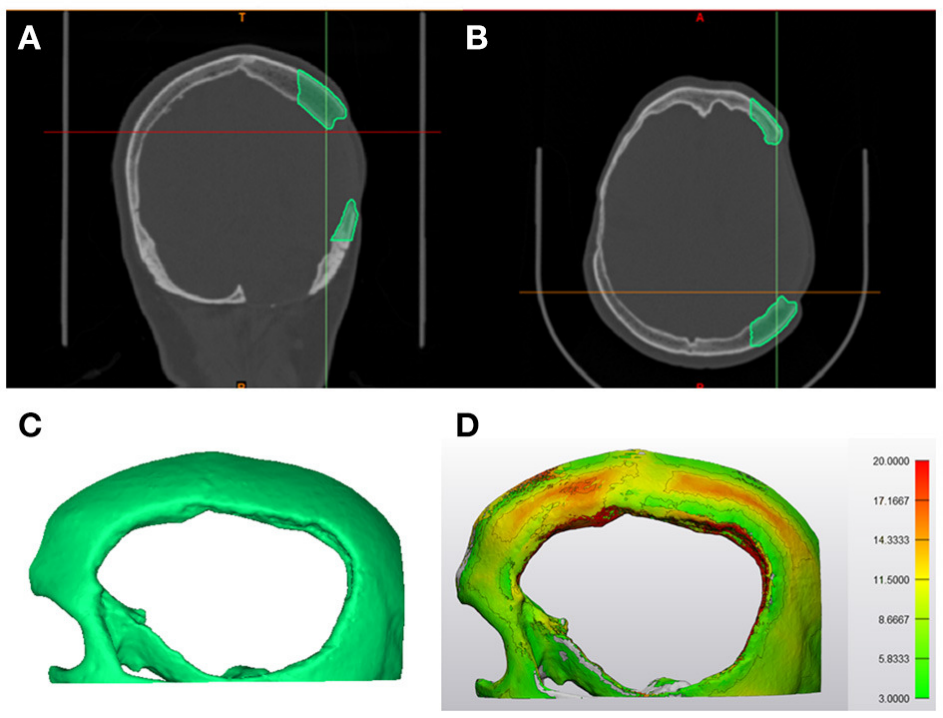
Steps involved in the generation of topographic bone thickness map. **(A)** Coronal view representing the bone segmentation (green) of the region of interest in the computed tomography (CT) data. **(B)** Axial view illustrating the bone segmentation (green) of the region of interest in the computed tomography (CT) data. **(C)** Profile view of the three-dimensional (3D) volumetric reconstruction of the area of interest. **(D)** The segmented skull displaying the color-coded topographic bone thickness map (TBTM).

The temporoparietal region has variable zones of bone density. Therefore, to integrate fixation points in the cranial prosthesis design, a topographic bone thickness map (TBTM) was generated. The TBTM helps visualize the bone stock available at the defect region and provides an intuitive fixation screw placement (Guignard et al., 2013). To create the bone thickness map, the CT DICOM datasets were visualized in a medical image processing software (Mimics Innovation Suite v. 21.0, Materialise, Leuven, Belgium). The crop mask (bounding box) functionality was used to select the region of interest, incorporating the bone around the defect area (Figures 4A,B). The bone region within the bounding box was segmented using thresholding. The smart fill functionality was used, and a 3D volumetric reconstruction of the skull defect was generated (Figure 4C). The TBTM was generated using the thickness analysis module (3-matic Medical v. 13.0, Materialise, Leuven, Belgium), resulting in a color-coded map. This surface-distance algorithm calculated the Euclidian distance for each point on the surface. A threshold level was set so that the bone portions with a thickness below 3 mm were excluded (Figure 4D). Subsequently, the quadruple fixation method with proximal and distal fixation points was integrated into the cranial prosthesis design.

## Results

### Customized Cranial Prostheses Wall/Strut Thickness Analysis

**Figure 5:**
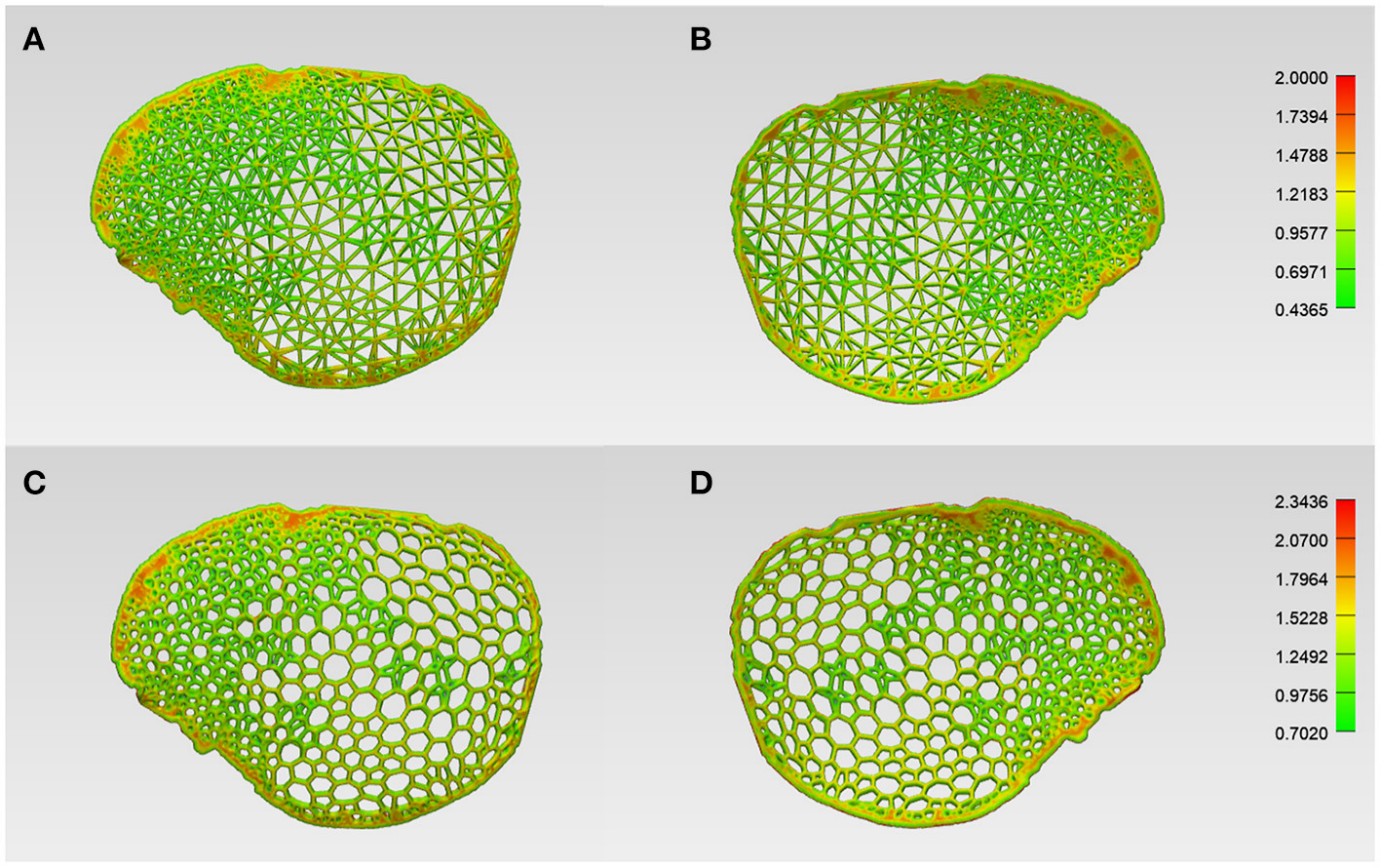
Color-coded maps illustrating the wall/strut thickness of the respective designs of the customized cranial prosthesis. **(A)** Wireframe/lattice Voronoi pattern prosthesis squamous (external) view. **(B)** Wireframe pattern prosthesis cerebral (internal) view. **(C)** Organic Voronoi pattern prosthesis squamous (external) view. **(D)** Voronoi pattern prosthesis cerebral (internal) view.

Before 3D printing, the patient-specific cranial prostheses' designs were analyzed for wall/strut thickness. The color-coded thickness map generated for the wireframe pattern prosthesis revealed that the implant had a range of variable thicknesses. The minimum and maximum strut thickness values were 0.43 and 2.00 mm, respectively, with a mean (SD) value of 0.88 (0.21) mm (Figures 5A,B). On the other hand, the organic Voronoi pattern prosthesis had a minimum and maximum strut thickness value of 0.70 and 2.34 mm, respectively, with a mean (SD) of 1.27 (0.21) mm (Figures 5C,D). In both designs, the maximum thickness was located at the peripheral aspect of the cranial prosthesis.

### Morphological Assessment of Virtual Reconstruction Fit and Contours

**Figure 6:**
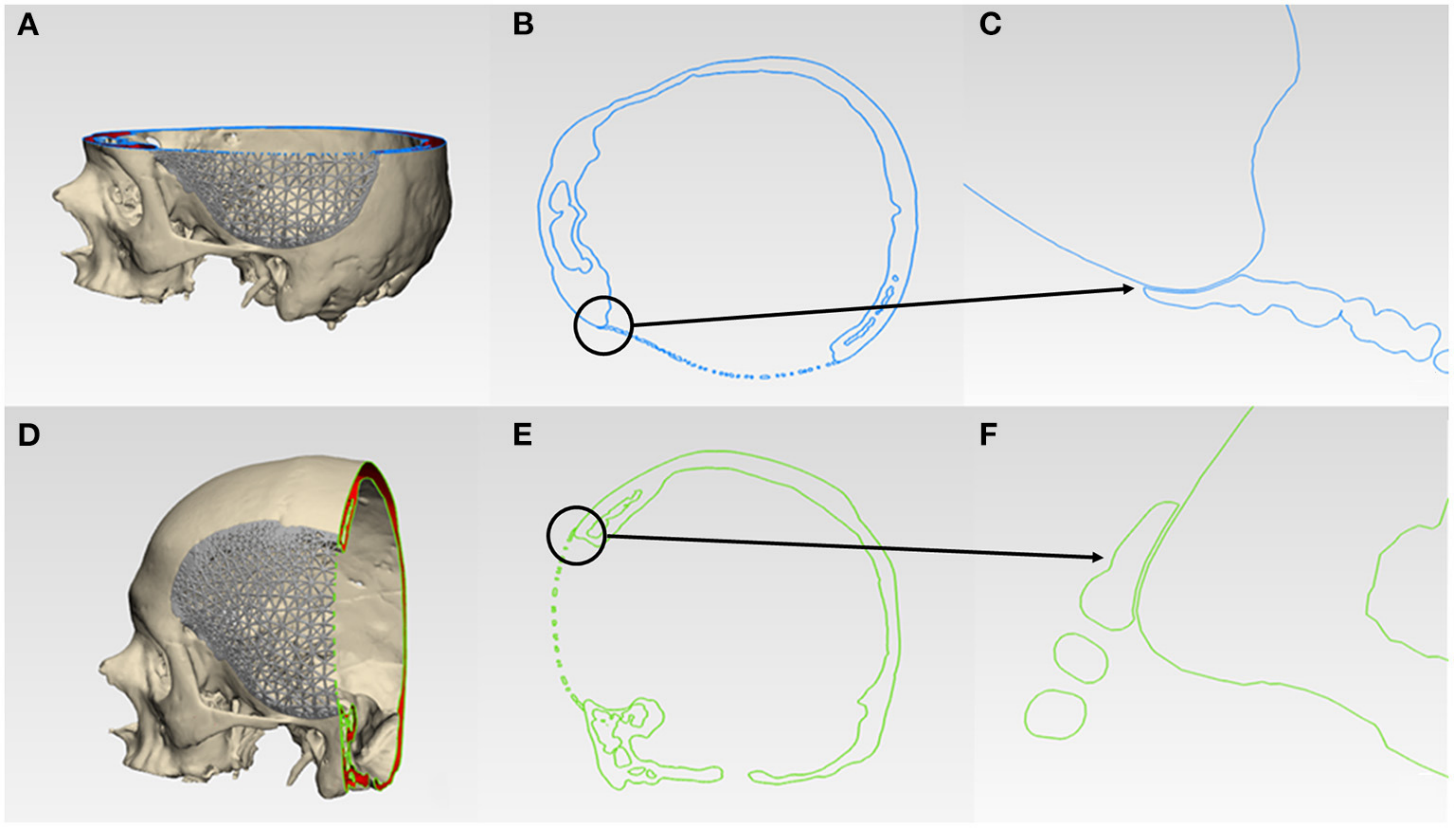
Virtual morphological fit and contour continuity analysis of wireframe/lattice Voronoi pattern customized cranial prosthesis. **(A)** Cutting plane in the Z-direction (blue). **(B)** Cross-sectional analysis along the z-axis. **(C)** Magnified view of the cross-sectional analysis along the z-axis. **(D)** Cutting plane in the Y-direction (green). **(E)** Cross-sectional analysis along the y-axis. **(F)** Magnified view of the cross-sectional analysis along the y-axis.

**Figure 7:**
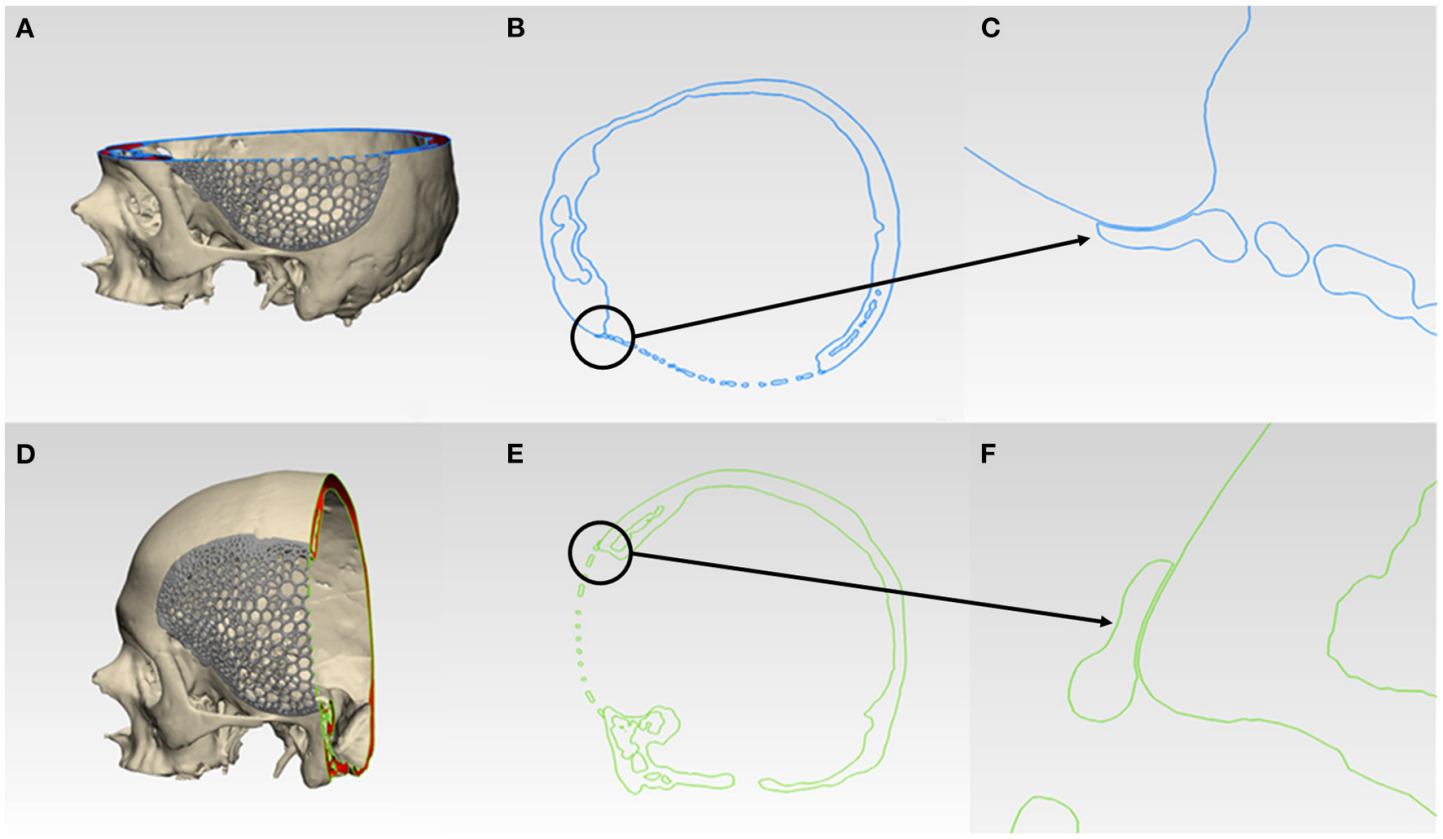
Virtual morphological fit and contour continuity analysis of organic Voronoi pattern customized cranial prosthesis. **(A)** Cutting plane in the Z-direction (blue). **(B)** Cross-sectional analysis along the z-axis. **(C)** Magnified view of the cross-sectional analysis along the z-axis. **(D)** Cutting plane in the Y-direction (green). **(E)** Cross-sectional analysis along the y-axis. **(F)** Magnified view of the cross-sectional analysis along the y-axis.

For morphological inspection, the virtual fit and the contour continuity between the designed implants and the skull were also examined. The .STL files of the skull model and the designed implants were analyzed in cross-sectional views in two perpendicular directions. Figures 6A,D and 7A,D illustrate the cutting planes in the y-axis and z-axis for the wireframe and organic Voronoi pattern prosthesis, respectively. The cross-sectional analysis results revealed good homogeneity of outer profile curvature contours between the skull model and the designed wireframe (Figures 6B,E) and Voronoi (Figures 7B,E) prosthesis. The tangency between the wireframe (Figures 6C,F) and Voronoi (Figures 7C,F) implants and the skull model was maintained, thereby revealing a satisfactory virtual reconstruction fit.

### Additive Manufacturing Processes for the Fabrication of Skull Biomodel and Biomimetic Patient-Specific Cranial Prostheses

The AM systems utilized in this case included two consecutive steps: (1) the fabrication of an anatomical biomodel using a Binder-Jetting (BJ) 3D printer; and (2) the fabrication of biomimetic patient-specific cranial prosthesis using a Selective Laser Melting (SLM) 3D printer. The biomodel was created to serve as a test to analyze the overall fit, congruence of the patient-specific cranial implants and further validate the virtual verification results.

The skull biomodel was manufactured using a BJ 3D printer (ProJet CJP 660Pro, 3D Systems, Inc., Rock Hill, USA). The selected materials were ZP 151 (powder gypsum) as the core material filling the build platform. An inkjet printhead then swept over the powder and selectively sprayed droplets of ZB 63 liquid binder agent or glue (3D Systems, Inc., Rock Hill, USA). The 3DPrint software v. 1.03 (3D Systems, Inc., Rock Hill, SC, USA) was used, and the biomodel was printed with a layer thickness of 100 μm using the default printer's settings. The unbound powder remained on the platform surrounding the biomodel, acting as support material. Once the entire first layer of the skull model was fused with the binder agent, the build platform moved down one layer in height, and a new layer of powder was deposited. This process was repeated until the fabrication of the skull biomodel was completed. The printed biomodel also called as “green body,” was encapsulated in the powder bed. The unbound, excess support powder was vacuumed and brushed away using pressurized air. The biomodel was subsequently infiltrated with an acrylic solution to achieve structural integrity.

The customized cranial prostheses were fabricated in Grade II titanium using SLM–a powder bed fusion 3D printing technology (SLM 250HL, SLM Solutions GmbH, Luebeck, Germany). The prototypes were fabricated with a layer thickness of 60 μm, using a YLR-Faser-Laser (200W), and the rest of the machine's settings were kept at default (argon flow, oxygen levels <0.1%, chamber temperature 200°C). The metal powder was spread across the build plate in layers by a coater blade. The whole process was carried out in an inert and thermally controlled environment using a high-power laser. A scanning mirror system directed the laser beam, and the powder material was heated to reach the melting point, thereby fusing the selectively melted powder material. When the material solidified, the build plate dropped down by one layer in height, and the coater spread another layer of fresh powder across the surface. The process was repeated until the fabrication of the prostheses was completed, followed by post-processing procedures.

**Figure 8:**
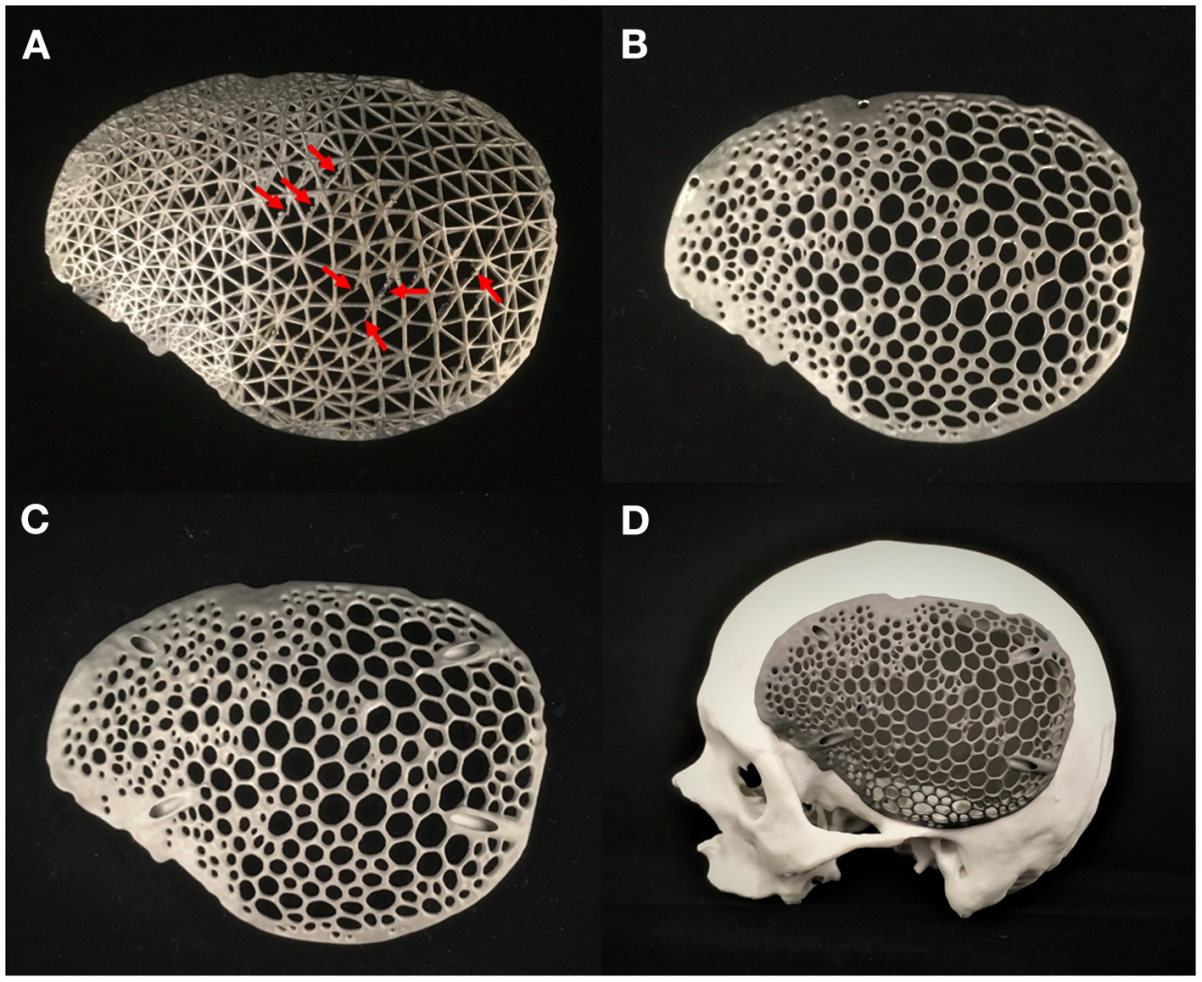
Additively manufactured skull biomodel and biomimetic patient-specific cranial prostheses. **(A)** Selective laser melting (SLM) 3D printed wireframe pattern prosthesis displaying structural printing issues (red-arrows). **(B)** Selective laser melting (SLM) 3D printed Voronoi pattern prosthesis with flange screw fixation points. **(C)** Selective laser melting (SLM) 3D printed Voronoi pattern prosthesis with angular screw fixation points. **(D)** Skull biomodel and customized cranial prosthesis illustrating precise anatomical fit.

The prototypes were used to access and verify the fabrication feasibility of the various designs. The cranial prostheses were printed in a vertical orientation with no support structures between the interconnected strut network. The wireframe/lattice pattern prosthesis had some regions of unfavorable printing results. Some struts had overhanging features requiring support structures. This caused residual stress build-up, resulting in structural printing issues (Figure 8A).

In contrast, the organic Voronoi pattern prosthesis revealed excellent printing feasibility. No deformation or defects were noticed in the fabrication of this design form. Two variants of screw fixation methods were designed in the organic Voronoi pattern cranial prosthesis—flange (Figure 8B) and angular fixation (Figure 8C). The biomimetic pattern prostheses were lightweight (30 g) and had an acceptable fit on the biomodel. Figure 8D illustrates the 3D printed skull biomodel and the SLM produced biomimetic cranial prosthesis after post-processing procedures.

### Dimensional Accuracy Assessment of the Biomimetic Patient-Specific Cranial Prosthesis

**Figure 9:**
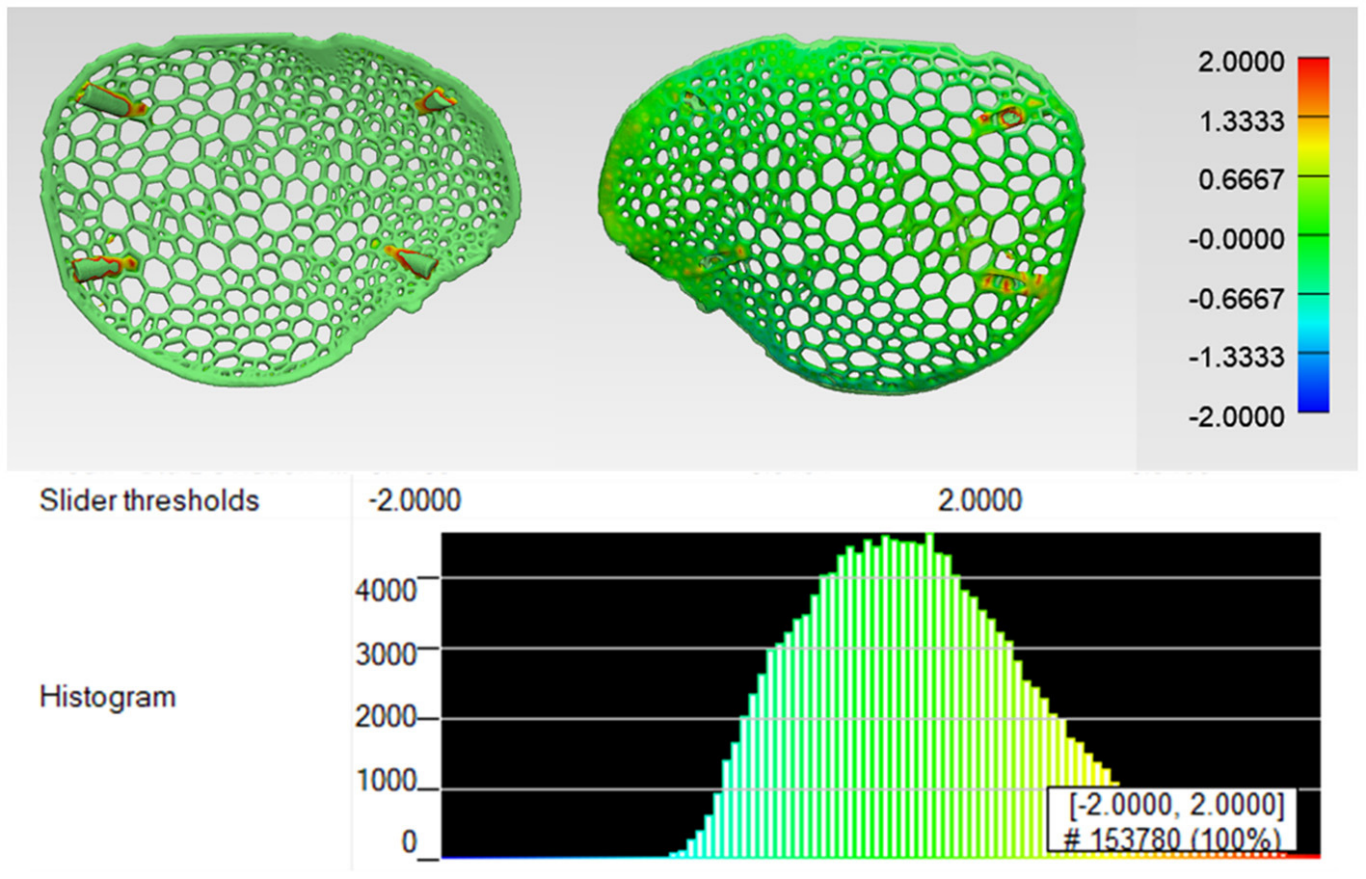
Dimensional accuracy assessment of biomimetic, organic Voronoi pattern prosthesis.

Due to structural printing defects in the wireframe pattern prosthesis (Figure 8A), the quantification of dimensional accuracy was estimated in organic Voronoi pattern prosthesis. The organic Voronoi pattern prosthesis was digitized using an optical-based scanning system (EinScan-SP, SHINING 3D Tech. Co., Ltd., Hangzhou, China); the generated 3D point cloud data was converted to an.STL file format. Using an ICP algorithm, a 3D part comparison analysis (3-matic Medical v. 13.0, Materialise, Leuven, Belgium) was carried out. The acquired point cloud was aligned with the reference (planned) prosthesis model using the best fit alignment protocol. The best fit alignment ensured that both the planned and printed prosthesis were positioned in the same coordinate system. A threshold of ±2 mm was set to accentuate the surface deviations, and a color-coded visual output was generated, where green represented high accuracy. The surface deviation analysis revealed the overall variations in root mean square error (RMSE). RMSE estimates how far the deviations are from 0, and a high value correlates to high deviations. Figure 9 illustrates the surface deviations in the positive and negative directions. The cranial prosthesis had high congruence (green-colored areas) with an overall RMSE value of 0.55 mm. Slight positive deviations (red-colored areas) were noticed around the screw fixations points.

## Discussion

Complex structures present in nature have inspired several domains such as visual arts, engineering, architecture, and most importantly, medicine. In parallel, computational power and computational methods have advanced adequately to facilitate the design of materials with complex structures tailored for specific applications (Vincent, 2009; Hwang et al., 2015; Schaedler and Carter, 2016). Unlike traditional manufacturing, AM offers novel avenues and has demonstrated the ability to fabricate complex structures with improved accuracy (Sing et al., 2016). The numbers of AM applications, especially in the medical implant manufacturing sector, have quickly increased and are expected to revolutionize health care (Emelogu et al., 2016; Willemsen et al., 2019; Fan et al., 2020; Ghilan et al., 2020). Previous studies have explored the use of CAD modeling and AM to improve both surgical planning and manufacture of customized implants with effective results (Jardini et al., 2014; Dodier et al., 2020; Tel et al., 2020). These advancements in CAD and AM technologies are gradually leveraging the innovation in craniomaxillofacial reconstructive surgeries.

The progress in the use of AM in personalized medicine has significantly raised interest in metal implants. The difference in the elastic modulus between the metal implant and bone causes a stress distribution mismatch, resulting in a “stress-shielding” effect (Sumner et al., 1998). To address this effect, regular and irregular porous constructs have been explored. The apparent elastic modulus of a porous metal implant is better suited to the bone and prevents the prosthetic loosening and fracturing of the implant in clinical applications (Geetha et al., 2009). Porous constructs have a large surface area and good intra- and inter-connectivity, facilitating cell adhesion, migration, nutrient supply, and biological fixation (Heinl et al., 2008; Levine, 2008). At present, the computer-aided modeling method of regular porous constructs is based on a unit cell. Any modification in the unit cell periodic design configuration results in an overall change of the whole construct, and thus the pore shape and size distribution are difficult to control. Du et al. demonstrated the superiority of irregular or asymmetrical designed porous constructs mimicking the human bone tissue (Du et al., 2020). The architecture of biomimetic irregular constructs based on Voronoi tessellations is limited to porous orthopedic scaffolds and has gained considerable attention (Chen et al., 2020).

This work's main findings on patient-specific cranial titanium prosthesis feature two characteristics–the generation of biomimetic, lightweight macrostructures based on Voronoi tessellations and their quasi-self-supporting prosthesis fabrication feasibility by SLM technology. By our definition, nature-inspired designs are not based on the exact morphological copy of nature counterparts but, instead, on taking a scientific approach to unravel the design fundamentals suitable to the perspective of nature and technology. The design based on Voronoi tessellations with interconnected struts and multiple gradient holes brings potential benefits. The current design workflow resulted in an efficient generation of a biomimetic structure conforming to the anatomical cranial bone shape. Besides, the generation of random design patterns can be achieved with variable characteristics (strut thickness, size, and the number of holes). This can be explained by the fact that the interactive generative design algorithms allow the user to modify the input parameters in appropriate ranges. By adjusting the strut thickness and the respective distance between them, the user can control the strength-to-weight ratio, optimize impact absorbance, stiffness, thermal conductivity, and, most importantly, supply spaces for tissues to attach and proliferate (Helou and Kara, 2018; Chen et al., 2020). In the case of cranial implants, supplied orifices efficiently provide extradural hematomas prevention.

To satisfy the reconstructive surgical requirements, the manufacturing process also plays a fundamental role. The fabrication of Voronoi structures is currently hardly feasible with traditional manufacturing methods in contrast to AM. Our study results demonstrated the quasi-self-supporting manufacturability of intricate designs based on Voronoi tessellations using SLM technology. SLM is a versatile manufacturing technique catering to a broad spectrum of metallic alloys and has been used to produce fully dense permanent implants (Gokuldoss et al., 2017; Zhang et al., 2018; Fan et al., 2020). The organic Voronoi pattern prosthesis had easier processability and excellent printing outcomes than the wireframe/lattice pattern prosthesis, explained by the downfacing sharp and angled interconnected struts configuration in the wireframe design. Rounded corners are structurally more beneficial than sharp corners and reduce the probability of crack development instead of sharp corners.

One of the most challenging features to print in SLM printing are the downfacing structures with a minimum thickness requirement of 0.4 mm. The powder bed cannot adequately support the liquid metal due to capillary, turbulent fluid flow, and gravity, and therefore, the melt pool is prone to defects (Chen et al., 2017). These factors can result in a sag on the downfacing regions, which affects the dimensional accuracy. Besides, warping and distortions can happen on the unsupported and overhanging sections (Xiang et al., 2019; Charles et al., 2020), as noticed in some wireframe/lattice prosthesis areas. A standard solution to address this is the addition of support structures to assist printability. However, the need to fabricate and then remove support material potentially increases the material consumption and requires a significant amount of post-processing, especially in thin-walled lattice structures. Although automated depowdering AM systems are available, the generation and removal of support structures in the pre- and post-processing procedures are still a time-consuming manual intervention. Furthermore, support structures typically result in wasted feedstock material and have to be discarded. As AM processes have energy costs, adding support structure increases the print time, fuelling energy consumption (Jiang et al., 2018). Cumulatively, these factors have a significant impact on the overall production cost of an implant.

Several researchers have explored support structure improvement by optimal part orientation and algorithms for support structure optimization (Jiang et al., 2018). However, the existence of support structures becomes problematic in structures with interior orifices and cavities. To overcome the problem as mentioned above, the design aspects become crucial. The generated structures are self-supporting, with surfaces having an overhang-angle smaller than a prescribed maximum overhang-angle. In our results, the quasi-self-supporting fabrication feasibility was supported by the organic Voronoi pattern prosthesis with high dimensional accuracy. The fabrication of implants without supports or a minimal number of support structures is imperative to reduce the manufacturing time, material consumption and improve the finishing processes. The analysis of the prosthesis's overall fit and congruence was assessed on a skull biomodel fabricated by BJ technology. BJ is a form of material jetting printing process and is very well-suited for anatomical biomodels requiring high accuracy (Ziaee and Crane, 2019; Msallem et al., 2020). Furthermore, multi-color biomodels can be fabricated with ease. The process is fast and affordable as it does not need support structures (Salmi, 2021).

Biomimetic designs' manufacturing depends on the AM technology, the selected material, and the overhanging angle that a 3D printer can support. To further comprehend these aspects, the manufacturing of the two cranial prosthesis designs was assessed by printing in Polyetheretherketone (PEEK)—a high-performance, biocompatible, thermoplastic biomaterial, using Fused Filament Fabrication (FFF) technology. We found that the fabrication of quasi-self-supporting, biomimetic patterned PEEK cranial plates was impossible with the FFF technology. After slicing in the PEEK FFF printer (M220, Apium, Additive Technologies GmbH, Karlsruhe, Germany), the biomimetic designs generate many segregated islands in each layer. The generated g-codes were extremely challenging to print on the FFF PEEK system, where low overhang angles, continuous filament deposition, and support structures are crucial fabrication prerequisites.

Moreover, there are challenges in designing infill patterns for continuous filament deposition. In PEEK FFF, material deposition is not possible “in the air,” and filament can only be deposited on an already solidified layer. Although angled walls can be fabricated, there is a limit to the maximum overhang angle (around 45°). Furthermore, for quality prints, the FFF material-extrusion printing process should be interrupted to a minimum. Each mid-course printing interruption changes the printing speed and results in a deposition defect affecting the fabricated object's dimensional characteristics. Consequently, most PEEK FFF patterns are comprised of continuous material deposition lines (Sharma et al., 2020). Due to these conditions, the Voronoi tessellations-based designs are more conducive for powder-based AM technologies, which have constraints different from FFF. For this reason, it is imperative to consider the additive manufacturing capabilities and follow the principles for AM design.

The Voronoi design titanium cranial implants were lightweight (30 g), required less material and fabrication time. A reduced manufacturing time is essential for PSIs, as it may reduce the waiting time for surgery. For biomedical titanium implants in general, an inherent structural problem of a solid/full-thickness implant is the stress-shielding phenomenon caused at the implant-bone interface, which can further lead to impaired bone healing (Parthasarathy, 2014; Li et al., 2020). Another potential problem of a solid titanium prosthesis is that it can be impervious to fluids. Some researchers have tried to reduce the stress shielding effect by changing the design constructs from solid to mesh-like porous reconstruction titanium plates (Chen et al., 2006). Although the meshed plates are lighter in construct, they are prone to deformation, especially in large-sized cranial deformities (De Water et al., 2016). Besides, the mesh plates need intraoperative manual bending, and any mismatch between the implant-bone interface might result in implant failure, thereby leading to a high revision rate (De Water et al., 2016). To overcome these problems, multiple holes/openings in the fabricated Voronoi design based PSIs replicated the feature of perforated plates. Being non-deformable, these PSIs maintain the cranial implant's structural integrity and provide adequate drainage of blood and cerebrospinal fluid-a preventive measure for hematomas and intracranial hypertension. These openings can also serve as dura suspension points on the cerebral side and temporalis muscle fixation points on the squamous side of the cranial prosthesis (Winston, 1999). Although titanium has excellent biocompatibility, bulk or solid titanium cranial prosthesis could additionally lead to high thermal sensitivity inside the body when exposed to very low or high temperatures. Therefore, an organic Voronoi design prosthesis can reduce thermal conductivity compared to a full-thickness, solid titanium implant.

While titanium cranial implants are less likely to deform during impact loading, these implants may break the surrounding bone structures. An alternative approach to mitigate this effect would be to use overlapping margins between the cranial prosthesis and the skull bone (Nout and Mommaerts, 2018). These overlapping margins designed during the preoperative planning phase can provide maximum contact at the implant-bone interface and help disperse the strain more efficiently. Another aspect that can be taken into consideration during the design phase is the screw fixation points. Titanium cranial prosthesis can suffer fixation screw pull-out, especially in the regions where strain is concentrated. The overlapping margins and angular fixation points can provide a more rigid fixation (Nout and Mommaerts, 2018).

The current workflow represents the integration and implementation of medical image data processing, design modeling, and AM technologies. These technologies can produce biomimetic customized cranial prosthesis with a perfect anatomical fit, conforming to the skull contours, thereby providing brain protection, faster functional recovery, and an esthetic rehabilitation with symmetrical cranial reconstructions. Although the application of Voronoi tessellations in the design and manufacture of titanium implants has a broad future, however, to increase the use of these design forms in medical implants substantially, several challenges need to be addressed. The fatigue life of Voronoi designed implants is still uncertain in the current research. Therefore, further tests on fatigue life should be carried out under the guidance of this design. Along with mechanics experiments, the *in-vitro* and *in-vivo* experimental validation is lacking for the newly designed structures. Additionally, the limitations of AM technology need to be considered. The error and printing accuracy of different technologies need to be evaluated to manufacture complex biomimetic structures.

Currently, biomimetic generative design is in an early developmental stage, which means defining a design problem in computable terms compatible with AM functionalities has certain limitations. First, as biomimetic generative design approaches can be computationally intensive and require hardware with powerful computing capabilities, software with cloud-computing capabilities can be developed. Second, with further advancements in artificial intelligence and machine learning, efficient and robust algorithms can be applied to the implant components combining finite element analysis solver in an iterative 3D design process. This will allow further optimization of the strut packing in an implant according to a multi-faceted objective function. Third, integrating generative design software with the respective AM 3D printer can assist in implant designing workflows. This will provide an assessment of whether an efficient and optimized design can be efficiently fabricated.

## Conclusion

Using generative design CAD modeling and SLM technology, we validated a new cranial implant's design and production feasibility. This concept provides a predictable approach to the design of a complex macrostructure mimicking bone trabeculae based on Voronoi tessellations. This biomimetic architecture of interconnected struts and multiple gradient holes has promising potential found on the discussed logical advantages, but further works for mechanical characterization and *in-vivo* experiments are needed to assess the clinical implications. Although the proposed work focussed on cranial prosthesis—a non-load bearing implant, the workflow opens the door to new intuitive 3D printed implants which can be extrapolated to other domains of medical implant manufacturing -orthopedics, cardiothoracic, spinal surgery, thereby expanding the role of Voronoi tessellations in biomimetic, patient-specific therapeutical solutions.

## Data Availability Statement

The original contributions presented in the study are included in the article, further inquiries can be directed to the corresponding author.

## Author Contributions

NS contributed to the conceptualization and writing of the original draft. DO, HR, PB, and FT contributed to the discussion, reviewing, and editing the manuscript. NS and DO contributed to design modeling, investigation, and validation. HR and FMT contributed to the project administration and supervision. FMT contributed to the resources. All authors conceived, discussed, read, and approved the final version of the manuscript.

## Conflict of Interest

The authors declare that the research was conducted in the absence of any commercial or financial relationships that could be construed as a potential conflict of interest.

## Abbreviations

2D: Two-Dimensional
3D: Three-Dimensional
AM: Additive Manufacturing
BJ: Binder Jetting
CAD: Computer-Aided Design
CAM: Computer-Aided Manufacturing
CT: Computer Tomography
DICOM: Digital Imaging and Communications in Medicine
FFF: Fused Filament Fabrication
HU: Hounsfield Units
ICP: Iterative Closest Point
PEEK: Polyetheretherketone
PSIs: Patient-Specific Implants
RMSE: Root mean square error
SD: Standard Deviation
SLM: Selective Laser Melting
STL: Standard Tessellation Language
TBTM: Topographic Bone Thickness Map
TPS: Thin-Plate Spline.
